# Identification and Biosynthesis of DHN-melanin Related Pigments in the Pathogenic Fungi *Monilinia laxa*, *M. fructicola,* and *M. fructigena*

**DOI:** 10.3390/jof9020138

**Published:** 2023-01-19

**Authors:** Lucía Verde-Yáñez, Núria Vall-llaura, Josep Usall, Neus Teixidó, Èlia Torreblanca-Bravo, Rosario Torres

**Affiliations:** IRTA, Postharvest Programme, Edifici Fruitcentre, Parc Científic i Tecnològic Agroalimentari de Lleida, Parc de Gardeny, 25003 Lleida, Spain

**Keywords:** brown rot, melanogenic genes, nectarines, secondary metabolism, stress conditions, survival

## Abstract

*Monilinia* is the causal agent of brown rot in stone fruit. The three main species that cause this disease are *Monilinia laxa*, *M. fructicola*, and *M. fructigena*, and their infection capacity is influenced by environmental factors (i.e., light, temperature, and humidity). To tolerate stressful environmental conditions, fungi can produce secondary metabolites. Particularly, melanin-like pigments can contribute to survival in unfavorable conditions. In many fungi, this pigment is due to the accumulation of 1,8-dihydroxynaphthalene melanin (DHN). In this study, we have identified for the first time the genes involved in the DHN pathway in the three main *Monilinia* spp. and we have proved their capacity to synthetize melanin-like pigments, both in synthetic medium and in nectarines at three stages of brown rot development. The expression of all the biosynthetic and regulatory genes of the DHN-melanin pathway has also been determined under both in vitro and in vivo conditions. Finally, we have analyzed the role of three genes involved in fungi survival and detoxification, and we have proved that there exists a close relationship between the synthesis of these pigments and the activation of the *SSP1* gene. Overall, these results deeply describe the importance of DHN-melanin in the three main species of *Monilinia*: *M. laxa*, *M. fructicola,* and *M. fructigena*.

## 1. Introduction

The genus *Monilinia* belongs to the Sclerotiniaceae family and is responsible for the brown rot that affects stone fruits, mainly peaches, nectarines, cherries, apricots, and almonds, although it can also affect pome fruits [1]. This pathogen can infect fruits during all fruit development, when most control strategies are applied, although the main losses of fruit occur at postharvest.

Plant diseases occur because of the timely combination of several elements; a susceptible host and a virulent pathogen contact and interact in a proper environment over a period. Previous studies have already shown how environmental conditions (i.e., temperature, humidity, and light) affect the development of *Monilinia* spp. and how these fungi express different phenotypes in response to environmental changes [2,3].

Fungi produce a wide variety of natural products that are not essential for growth and development, but can contribute to survival under stress conditions such as drought, extreme temperatures, and ultraviolet (UV) radiation [4]. One way that the pathogen is able to tolerate environmental stress conditions is through the production of secondary metabolites such as pigments, and particularly melanin. Melanin is broadly defined as a brown to black macromolecule with great diversity and structural complexity, derived from oxidative polymerization of phenolic precursors [5,6]. The melanin structure is resistant to acid hydrolysis but susceptible to degradation under alkaline conditions with NaOH [7]. Currently, melanin can be classified into eumelanins, pheomelanins, neuromelanins, and allomelanins [8]. The eumelanins contain nitrogen atoms, pheomelanin and neuromelanins contains nitrogen and sulfur atoms while allomelanins contain neither [9].

Fungal melanin performs multiple biological functions, contributing to survival under stress conditions and improving the competitive capabilities of the species. In many organisms, such as *Magnaporthe grisea* and *Botrytis cinerea*, melanization has been described as a protection process against damage caused by UV radiation [10]. In other fungi, such as *Sclerotinia sclerotiorum* and *Verticillium dahliae*, melanin production also plays a role in virulence, contributing to the development of specialized structures in the infection process to improve their resistance to the host [5,10].

Fungi can biosynthesize melanin by two different pathways: either through the L-3,4 dihydroxyphenylalanine (L-DOPA) pathway, which is found primarily in basidiomycetes [11] and human pathogens [12], or through the 1,8-dihydroxynaphthalene (DHN) pathway, which is usually found in ascomycetes [13,14], such as fungi belonging to the Sclerotiniaceae family. It is believed that the L-DOPA pathway is spontaneously activated after initiating the oxidation by a laccase [15]. On the other hand, some fungi belonging to the Sclerotiniaceae family, such as *B. cinerea*, uses malonyl-CoA/acetyl-CoA as a precursor of the DHN pathway, which serve as initiator and extender units of polyketide synthase (PKS) which catalyze the first step in the biosynthetic pathway, being the key gene for melanin biosynthesis. In *B. cinerea*, both *PKS12* and *PKS13* are initiators of DHN-melanin, producing 1,3,6,8-tetrahydroxynaphthalene (T4HN), although by different pathways. The T4HN compound is reduced to scytalone by a first reduction reaction produced by THNs reductases (*BRN1* and *BRN2*). Scytalone forms 1,3,8-trihydroxynaphthalene (T3HN) through a dehydration reaction carried out by scytalone dehydratase (*SCD1*). A second reduction reaction produced by THNs reductases (*BRN1* and *BRN2*) forms the vermelone compound from T3HN, which is converted by scytalone dehydratase (*SCD1*) into the final product, DHN-melanin [12]. Melanin polymers formed by the oxidative polymerization of DOPA, DHN, or similar substrates contain mixtures of quinone, hydroquinone, and semiquinone in the polymers. These structures explain the characteristic absorption of light by melanins [7].

In the case of *Monilinia* spp., little information is available on melanin biosynthesis. In *M. fructicola*, melanin-deficient mutants have been isolated and characterized, suggesting that melanin provided conidia with resistance to a variety of environmental stresses [16], and required the DHN-melanin pathway for tolerance to osmotic stress and virulence [17]. In another study performed by Villarino et al. [18], the results showed that the ability of *M. laxa* to produce melanin is crucial for its pathogenicity. Yu et al. [19] have identified by gene silencing the gene *MfPKS12* as one of the precursors of the DHN-melanin pathway that contributes to DHN-melanin accumulation and virulence in *M. fructicola*. However, the mechanism underlying melanin production and the description of the DHN-melanin biosynthetic machinery in *M. laxa* and *M. fructigena* have not yet been studied.

To contribute to the enlightenment on the production of melanin and the biosynthesis of DHN-melanin by *Monilinia* spp., in this study, we have quantified the melanin-like pigments produced by *M. laxa*, *M. fructicola,* and *M. fructigena* grown under darkness in in vitro conditions, and in infected nectarines at different brown rot developmental stages (S1: peel surface maceration stage; S2: colonization stage; and S3: spreading stage). Moreover, we have identified the melanin biosynthetic machinery derived from the DHN pathway in the genome of these species, and we have analyzed their gene expression in both growing conditions (in vitro and in nectarines), providing, for the first time, a complete overview of the DHN-melanin biosynthesis on the pathogenic fungi *M. laxa*, *M. fructicola*, and *M. fructigena*.

## 2. Materials and Methods

### 2.1. Fungal Material and Culture Conditions

The species of *Monilinia* used in this study were *M. laxa* (ML8L), *M. fructicola* (CPMC6), and *M. fructigena* (GENA6). *Monilinia laxa* and *M. fructicola* were deposited in the Spanish Culture Type Collection (CECT 21100 and CECT 21105, respectively) and the Bioproject code for *M. fructigena* is PRJNA707424.

Conidial suspensions were prepared from 7 9-day-old cultures grown on potato dextrose agar supplemented with 25% tomato pulp (PDA-T). The inoculum of *M. laxa* and *M. fructicola* species was prepared as described by Baró-Montel et al. [20]. Due to the inability of *M. fructigena* to produce conidia, the fungal suspension was obtained as described by Verde-Yáñez et al. [3]. Suspensions of three species of *Monilinia* were inoculated in PDA-T medium for 7 days under darkness at 20 °C. Finally, fungal colonies of *Monilinia* spp. were frozen to quantify melanin-like pigments and gene expression analysis (qPCR). All experiments were performed twice using at least three replicates per condition.

### 2.2. Plant Material and Fruit Inoculations

‘Red Jim’ nectarines (*Prunus persica* (L.) Batch) were harvested at commercial maturity from an organic orchard located in Lleida (Catalonia, Spain). Fruit was homogenized based on the single index of absorbance difference (DA index) using a portable DA-Meter (TR-Turoni, Forli, Italy), comprising a DA index between 0.08 to 1.58.

Nectarines were inoculated with a suspension of conidia of each *Monilinia* spp. (*M. laxa*, *M. fructicola* and *M. fructigena*), by applying six drops of 10 μL of 10^5^ conidia mL^−1^. Fungal suspensions of each pathogen were obtained as explained in Section 2.1. The inoculated fruit was incubated in a chamber under darkness and a relative humidity of 97 ± 3% at 20 ± 1 °C. Three different brown rot developmental stages were selected: peel surface maceration stage (S1), colonization penetration stage (S2), and spreading stage (S3). For both *M. laxa* and *M. fructicola*, the different stages corresponded to 14-, 24-, and 72-h post-inoculation (hpi), respectively. For *M. fructigena*, the different stages corresponded to 72 hpi (S1), and to 4 and 5 days post-inoculation (dpi) for S2 and S3, respectively. Six cylinders of peel and pulp tissue (1 cm diameter and depth) encompassing the inoculation sites were sampled from each fruit and pooled for each replicate at each sampling point. Samples were immediately frozen in liquid nitrogen and stored at −80 °C to quantify melanin-like pigments and gene expression analysis (qPCR). Four replicates of five fruits were obtained for each time point and species. For disease symptom visualization, images of the inoculated fruit at different stages were taken.

### 2.3. Extraction and Quantification of Melanin-like Pigments Produced by Monilinia spp.

The extraction of melanin-like pigments from *Monilinia* spp. was carried out as described by Villarino et al. [18], with some modifications. Briefly, a total of 150 mg of both in vitro samples (fungi) and infected nectarines (containing both fungal and fruit tissue) for each species was weighed, added to 10 mL of sterile water, and incubated in a water bath at 98 °C for 15 min. Subsequently, samples were centrifuged to recover the pellet and washed twice with sterile water. The pellet obtained was autoclaved with 3 mL of 1 M NaOH to recover thereafter the supernatant, which was acidified with 35% HCl. The supernatant was centrifuged and incubated in a water bath at 80 °C for 3 h. Then, the precipitate obtained was centrifuged, washed with sterile water, and dried at 100 °C for 1 h. Finally, 2 mL of 2 M NaOH were added to the dry extract, and incubated for 15 min at 50 °C. The melanin-like content was determined by spectrophotometry (UV-1100, dDBioLab, SLU) at OD_414_, using a standard curve with synthetic melanin (Sigma, St. Louis, MO, USA). Melanin quantification of both in vitro samples and infected nectarines samples was performed twice using three replicates per condition.

### 2.4. Identification of DHN Biosynthetic and Regulatory Genes in Monilinia spp.

A BLAST analysis was performed to identify the melanogenic genes of the DHN-biosynthetic pathway in the genome of the three *Monilinia* species using *B. cinerea* as the reference genome (Supplementary Table S1). Candidate genes from *B. cinerea* were used as query sequences for a BLAST analysis to search for homologies within *M. laxa* (ML8L) [21], *M. fructicola* (CPMC6) [22], and *M. fructigena* (GENA6) [23] genomes using NCBI Genome Workbench software v. 2.11.10 (https://www.ncbi.nlm.nih.gov/tools/gbench/ (accessed on 22 November 2022)), and the BLAST tool implemented therein, setting an expect (E) value of 10^3^. The identity (>60%) and the fraction of query sequences covered by the match region (>50%) were used as filter criteria to select only reliable hits. Results obtained were checked by carrying out BLATSX, BLASTN, and TBLASTN analyses.

The genomic positions of the melanogenic genes for each species were evaluated using the Integrative Genomics Viewer (IGV) program (v. 2.15.0, CA: University of California). The InterPro databases EMBL-EBI (https://www.ebi.ac.uk/interpro/ (accessed on 5 December 2022)) were used to identify the protein domains for transcription factors (TFs) of the DHN-melanin pathway.

### 2.5. Fungal RNA Extraction and qPCR Analysis

Total RNA extraction corresponding to the in vitro samples was extracted using TRI reagent (Sigma, St. Louis, MO, USA) as described by Baró-Montel et al. [20], using 3 biological replicates for each species. Total RNA from ‘Red Jim’ infected nectarines was extracted following the protocol described by Baró-Montel et al. [24]. Total extracted RNA was DNase-treated and quantified using a ND-1000 NanoDrop spectrophotometer (Thermo Scientific, Wilmington, DE, USA). The RNA quality was assessed by electrophoresis on an agarose gel stained with GelRedTM Nucleic Acid Gel Stain (Biotium, Hayward, CA, USA).

cDNA synthesis was performed on 5 µg and 2 µg of total RNA for in vitro and for in vivo conditions, respectively, using the commercial Superscript IV First-Strand reverse transcriptase cDNA Synthesis Reaction kit (Invitrogen, Carlsbad, CA, USA).

Gene expression analysis was performed as described by Baró-Montel et al. [20], using KAPA SYBR^®^ Fast qPCR Master Mix (Kapa Biosystems, Inc., Wilmington, MA, USA) on the 7500 Real Time PCR System (Applied Biosystems, Waltham, MA, USA). Primers used for gene expression analysis of melanin biosynthesis and regulatory genes (Supplementary Table S2) were designed de novo using the Primer-BLAST tool [25], or retrieved from Verde et al. [3] in the case of *STE12*, *OPT1,* and *SSP1* genes. For each selected gene, primer sequences were common to the three *Monilinia* species. *ELONGATION FACTOR 1A* (*EF1-α*) was selected as reference gene based on its constant expression among conditions. Primer efficiency was determined using 3-fold cDNA dilutions in triplicate and primer specificity was checked by analyzing the melting curves at temperatures ranging from 60 to 95 °C. A non-template control (NTC) was included using water instead of DNA. Relative gene expression was expressed as mean normalized expression (MNE) and calculated using the method described by Muller et al. [26].

### 2.6. Determination of Fungal Biomass of Nectarines

The fungal biomass of nectarine samples infected with *Monilinia* spp. at different brown rot developmental stages (S1, S2, and S3) was determined by analyzing the expression of the *Monilinia* spp. reference gene *EF1-α* normalized to the expression of the nectarine reference gene *TRANSLATION ELONGATION FACTOR 2* (*TEF2)*, as detailed in the above Section 2.5.

### 2.7. Statistical Analysis

All data were collated and subjected to analysis of variance (ANOVA) using JMP^®^ 16 (v. 16.0.0, Cary, NC, USA: SAS Institute Inc.). When the analysis was statistically significant, Tukey’s HSD test was performed at the *p* ≤ 0.05 level for the comparison of means at different brown rot developmental stages (S1, S2, and S3), or among *Monilinia* species. Correlations between experimental variables were made using the Pearson’s product moment correlation (*p* ≤ 0.05). 

## 3. Results and Discussion

### 3.1. The Biosynthetic and Regulatory Genes of the DHN-melanin Pathway Are Present and Differently Clustered in the Genome of the Three Monilinia spp.

The BLAST analysis on the *Monilinia* spp. genome confirmed the existence of the DHN-melanin biosynthetic and regulatory genes in the three main species of *Monilinia* (Table 1). Overall, we obtained a sequence coverage close to 100% in the nine genes identified for the three *Monilinia* spp., and a sequence identity higher than 70%, revealing the high gene conservation among species. Therefore, these results suggest that they could have conserved functions with *B. cinerea* or even within the Sclerotiniaceae family. As an exception, *BROWN 1*(*BRN1)* gene showed only 27% identity with its orthologue in *B. cinerea* gene, although only in the *M. laxa* species.

**Figure 1:**
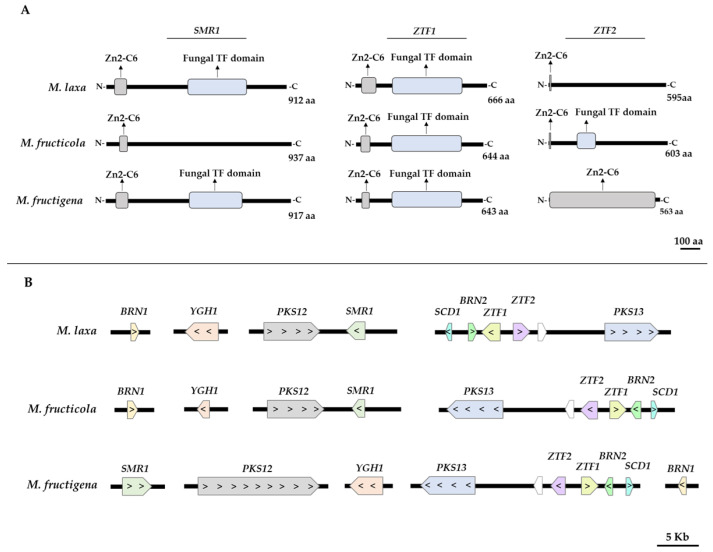
(**A**) Domain architecture of the regulatory genes *SMR1*, ZTF1, and *ZTF2* of the three *Monilinia* spp. involved in the DHN-melanin pathway. (**B**) Distribution of the DHN-melanin related genes in the genomes of *Monilinia* spp.

In *B. cinerea*, the genes *SCLEROTIAL MELANOGENESIS REGULATORY GENE- 1 (SMR1)*, *TRANSCRIPTION FACTOR 1* (*ZTF1),* and *TRANSCRIPTION FACTOR 2* (*ZTF2)* are considered transcription factors, regulating the genes involved in the DHN-melanin pathway in different conditions [12]. Accordingly, we inspected if in the case of *Monilinia* spp., these genes also contained the expected transcription factor (TF) domains (Figure 1A). The results revealed that in the three *Monilinia* spp., the three genes contained a fungal TF (IPR007219/IPR021858) or at least, a Zn2-C6 fungal type DNA-binding domain (IPR036864). Specifically, the *SMR1* gene contained the Zn2-C6 domain in the three species, although the fungal TF domain was only detected in *M. laxa* and *M. fructigena*. The *ZTF1* gene highlighted for containing both domains as also observed in the case of *B. cinerea* [12]. The same occurred for *ZTF2*; although, in the case of *M. laxa* and *M. fructigena*, it was only detected the Zn2-C6 fungal domain. The presence of at least one of these domains points out that these genes could be acting as regulatory genes as also observed for the closed organism *B. cinerea*. In other organisms such as *C. lagenarium,* it was shown that the Zn2-C6 domain was required for wild-type levels of melanization [27].

According to these results, we further analyzed in detail the position in the genome of the different biosynthetic genes, as well as the regulatory genes for each *Monilinia* spp. In many fungi, the genes related to the DHN-melanin pathway are clustered or partially clustered. Specifically, in the close relative *B. cinerea*, a partial clustering of the biosyntheticgenes of the DHN-melanin pathway, located on chromosomes 2, 3, and 4 and clustered to genes that encode the transcription factors *SMR1*, *ZTF1,* and *ZTF2,* was revealed [12]. In previous studies carried out with *A. fumigatus* [28], six genes of the DHN-melanin pathway (*ABR1*, *ABR2*, *AGY1*, *ARP2*, *ARP1*, *ALB2*) were also identified as partially clustered within a fragment of 19 kb DNA.

In the same line, our results demonstrated that the biosynthetic and regulatory genes of *Monilinia* spp. were also partially clustered in the genome (Figure 1B). The cluster organization and gene location were more similar in the case of *M. laxa* and *M. fructicola*, than in *M. fructigena* species. In both *M. laxa* and *M. fructicola*, the *POLYKETIDE SYNTHASE- 12 (PKS12)* gene was physically clustered to the *SMR1* TF, while in the case of *POLYKETIDE SYNTHASE- 13 (PKS13)* gene, it was physically clustered to both TFs (*ZTF1* and *ZTF2*) and *BROWN 2 (BRN2)* and *SCYTALONE DEHYDRATASE- 1* (*SCD1)* genes. These results were in line with that observed in *B. cinerea* [12]. As for the other species, the *M. fructigena PKS13* gene was also clustered with the TFs *ZTF1* and *ZTF2*, and with *BRN2* and *SCD1* genes. Interestingly, our results demonstrated that, differently to *M. laxa* and *M. fructicola*, the *SMR1* TF of *M. fructigena* was not physically clustered to the *PKS12* gene, suggesting that this gene is probably not under the control of *SMR1*, and that for this species, the regulation of the DHN-melanin pathway occurs differently from that of the other species of the same genus. These differences could, in part, ultimately explain the different fitness and infection/survival capacities of these species [29]. Finally, the *YELLOWISH-GREEN HYDROLASE- 1* (*YGH1)* and *BRN1* genes were not identified liked to other DHN-related genes in neither of the three *Monilinia* species. Their putative relationship with other genes not identified in this study should be further analyzed.

Overall, these results demonstrate, for the first time, that the three species analyzed have the machinery to synthetize melanin through the DHN pathway and that the regulatory mechanisms are largely conserved with other Sclerotiniaceae. The capacity to produce DHN-melanin probably relies on the *PKS12* and *PKS13* genes, a mechanism that would be shared with *B. cinerea* [12]. In fact, Schumacher et al. [12], by generating mutants, identified *PKS12* and *PKS13* as the key enzymes for melanin synthesis. However, the specific and interesting differences detected for *M. fructigena* should reveal a different regulatory mechanism that could explain the different capabilities of this species.

### 3.2. All Three Monilinia Species Produce Melanin-like Pigments

Once the presence of the DHN-melanin machinery in the three *Monilinia* spp. Was demonstrated, the next step was to analyze their ability to produce these pigments and the possible differences between species. Firstly, to check the maximum emission of melanin-like pigments, the synthetic melanin was scanned in a range of OD_400_ to OD_540_. The maximum emission occurred at OD_414_ nm in agreement with Villarino et al. [18].

**Figure 2:**
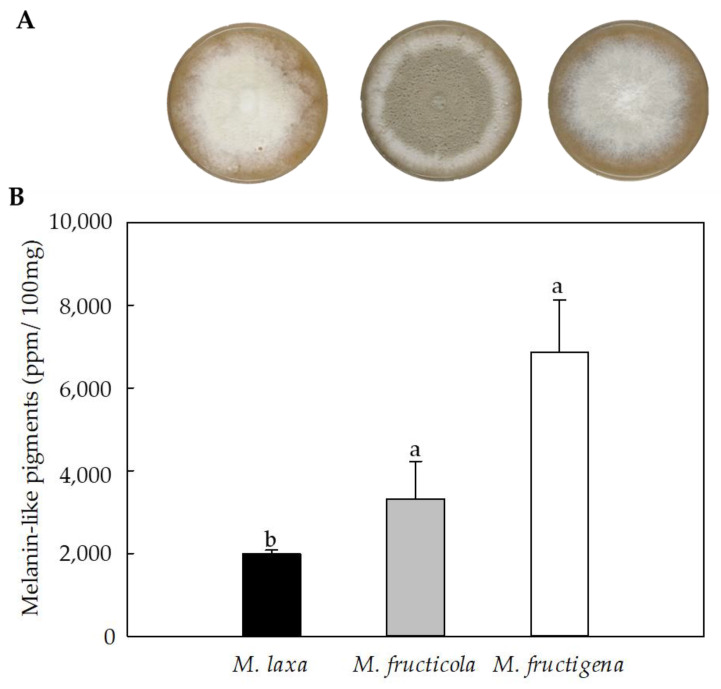
(**A**) In vitro phenotype of *M. laxa*, *M. fructicola*, and *M. fructigena* grown on PDA-T medium and incubated for seven days under darkness. (**B**) Spectrophotometric quantitation (OD_414_) of melanin-like pigments expressed as parts per million (ppm) in *M. laxa*, *M. fructicola,* and *M. fructigena* grown on PDA-T medium and incubated for seben days under darkness. Letters indicate significant differences (*p* ≤ 0.05) between species. The error bars represent the standard deviation of the means (n = 3).

Cultures of *Monilinia* spp. grown under darkness for seven days showed differences be-tween the species on the pigmentation of the colonies (Figure 2A). Specifically, *M. laxa* showed a white coloration, probably due to a high production of mycelium, characteristic of this species when grown under darkness. The phenotype of *M. fructicola* colony high-lighted for presenting an olivaceous color and a high content of conidia with a white peripheral growth ring. In the case of *M. fructigena*, the colony showed a uniform grey color, probably also due to its mycelial structures. Similar results were obtained in our previous study [3] based on the morphological characterization of *Monilinia* spp. under darkness and under different light wavelengths conditions.

The results obtained for the quantification of melanin-like pigments in *Monilinia* spp. showed that the three *Monilinia* species could produce melanin-like pigments under in vitro conditions (Figure 2B). The maximum emission of the samples occurred at OD_414,_ as did the synthetic melanin. Under in vitro conditions, *M. fructicola* and *M. fructigena* produced a similar production of melanin-like pigments. The highest production of melanin-like pigments was significantly higher in *M. fructigena* (3.45-fold) and *M. fructicola* (1.47-fold) than in *M. laxa*. These results were in line with the pigmentation of the colonies (Figure 2A), where the colorless phenotype was observed in *M. laxa*. No significant differences were obtained between *M. fructicola* and *M. fructigena* species. Although previous studies have demonstrated the capacity of *M. fructicola* [19] and *M. laxa* [18] to produce melanin-like pigments, no studies are yet available for *M. fructigena*. In several organisms, such as *Aspergillus fumigatus*, *A. nidulans* [30], *Magnapothe grisea* [31], and *Penicillium marneffei* [32], the capability of producing melanin was also demonstrated after being cultivated in both solid or liquid media.

The production of melanin-like pigments in fungi could be strongly related to environmental factors such as light, humidity, and/or temperature. Raman et al. [33] demonstrated the positive correlation between temperature and humidity and its direct dependence on fungal growth and melanin production. This could, in part, be attributed to the protective role of melanin in mitigating the stressful conditions caused by the exposition of the fungus to certain environmental situations.

### 3.3. Melanin Content along the Nectarine Infection Process Is Different among Monilinia spp.

**Figure 3:**
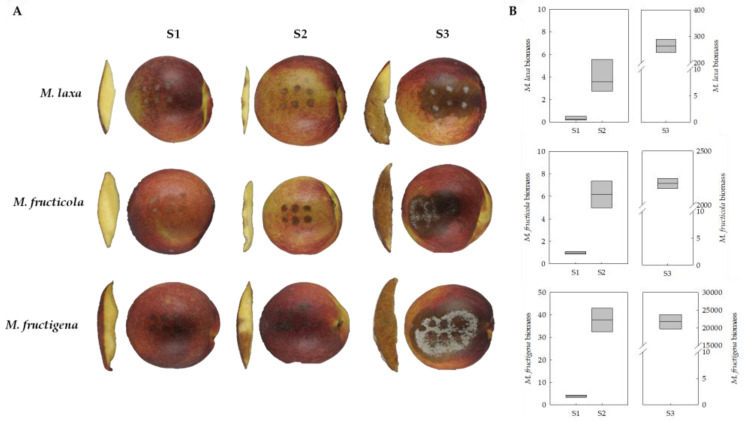
Brown rot spread in ’Red Jim’ nectarines at different stages after inoculation: peel surface maceration stage (S1), colonization of the pathogen (S2), and spreading stage (S3). (**A**) Two different viewpoints are shown (left image: perpendicular section of the fruit to discern fungal colonization if observable; right image: entire fruit showing 6 drops). (**B**) Determination of pathogenic biomass by relative gene expression of the reference gene of *Monilinia* spp. (*EF1-α*), normalized to the expression of the reference gene of nectarine (*TEF2*) for each *Monilinia* spp. The box plot represents the mean of three biological replicates with their interquartile range.

To assess the importance of melanin-like pigments on the pathogenic process of *Monilinia* spp., three stages of brown rot development were selected for monitoring the production capacity of melanin-like pigments of the three main species when infecting nectarines (Figure 3A). The first infection stage (S1) was selected coinciding with the peel surface maceration (peel color change) at the site of inoculation of the fruit peel. The second selected stage (S2) was determined by the colonization of the mesocarp caused by the pathogen. Lastly, the third sampling point (S3) covered the spreading of the disease through the fruit pulp and the production of either conidia (*M. laxa* and *M. fructicola*) or mycelium (*M. fructigena*) on the fruit surface. Moreover, and to determine the disease progress, the fungal biomass was estimated in the fruit inoculated with the three *Monilinia* species (Figure 3B). The results obtained showed that the expected fungal biomass of nectarines increased as the disease progressed, obtaining the highest biomass production in S3 for the three *Monilinia* species. At the S3 stage, the biomass of all fungi increased exponentially, the biomass of *M. fructigena* being higher than that for *M. fructicola* or *M. laxa*.

**Figure 4:**
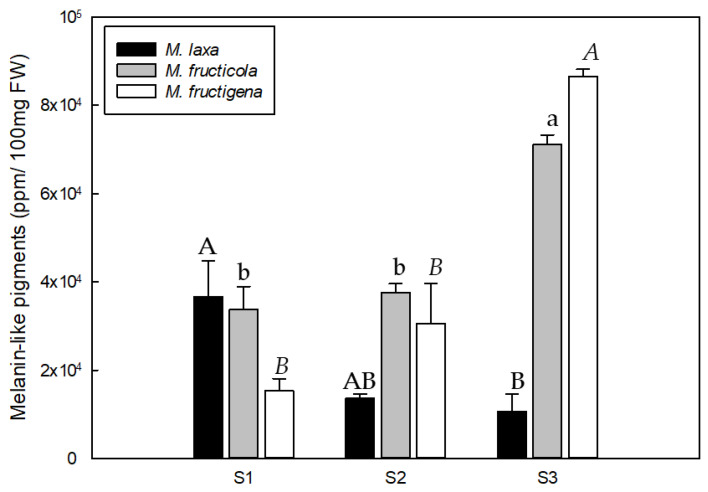
Spectrophotometric quantification (OD_414_) of *Monilinia* spp. melanin-like pigments expressed as parts per million (ppm) during the infection process of ‘Red Jim’ nectarines at different stages of the disease development (S1: peel surface maceration stage; S2: colonization stage; and S3: spreading stage) following incubation under darkness. Different uppercase, lowercase, and italic letters indicate significant differences (*p* ≤ 0.05) for *M. laxa*, *M. fructicola,* and *M. fructigena* species, respectively, between disease development stages. Error bars represent the standard deviation of the means (n = 3).

The quantification of melanin-like pigments of the three *Monilinia* spp. during the infection process of nectarines unraveled differences among brown rot developmental stages for all the species analyzed, and also a different production pattern along the infection course among species (Figure 4). In particular, *M. laxa* produced a higher content of melanin-like pigments in the first stage (S1), which was significantly higher (3.43-fold) than the last stage (S3). Therefore, the quantity of melanin-like pigments produced by *M. laxa* decreased with the development of disease, despite the higher fungal biomass in the last stage (S3) compared to the previous stages (S1 and S2). This indicates the important role of melanin in *M. laxa* during the early stages of infection (recognition and colonization). In contrast, the pattern of *M. fructicola* and *M. fructigena* was completely different since melanin-like pigments production increased with the development of the disease and was significantly higher at S3 in relation to S1 in both species. Specifically, in the case of *M. fructicola*, the production of melanin-like pigments did not significantly increase until the last stage (S3) of conidiation and the spreading of the fungus, during which it showed a greater production of conidia. This behavior was also observed in *M. fructigena*. Although the spreading stage (S3) was the one in which the highest content of melanin-like pigments was detected, *M. fructigena* was not able to generate conidia like *M. fructicola*, demonstrating that, for this species, the pigmentation is mostly due to the grey color produced during the vegetative growth. Hence, and unlike *M. laxa*, the melanin-like pigments in both *M. fructicola* and *M. fructigena* would be more related to the survival and spreading of the disease, rather than to its initiation. Although some studies point out to the key role of melanin in the development of appressoria [19], our results demonstrated that both *M. fructicola* and *M. fructigena* seemed to not require melanization for the successful infection of the host fruit. These results are in line with the previous studies from Rehnstrom et al. [16], who demonstrated that melanin-deficient mutants of *M. fructicola* are able to produce conidia, though they are less resistant to environmental stresses. In fact, melanin can enhance the survival and competitive abilities of organisms in certain environments; however, to date, they have been demonstrated as non-essential for growth and development. On the other hand, the production of melanin-like pigments by *M. laxa,* mainly during the first stage of the infection, points to its crucial role for the pathogenicity of this species. Overall, demonstrates for the first time the different putative role that melanin plays in the three main species of *Monilinia*, implying a first step towards the better understanding of this complex disease. Although the differences between species and stages are evident, the role of fruit polyphenol oxidases in the generation of melanin through quinones (Mi Moon et al. [34]), should not be obviated.

### 3.4. The DHN-melanin Biosynthetic Machinery Is Activated in Monilinia spp., Dependent on the Species and on the Disease Stage

**Figure 5:**
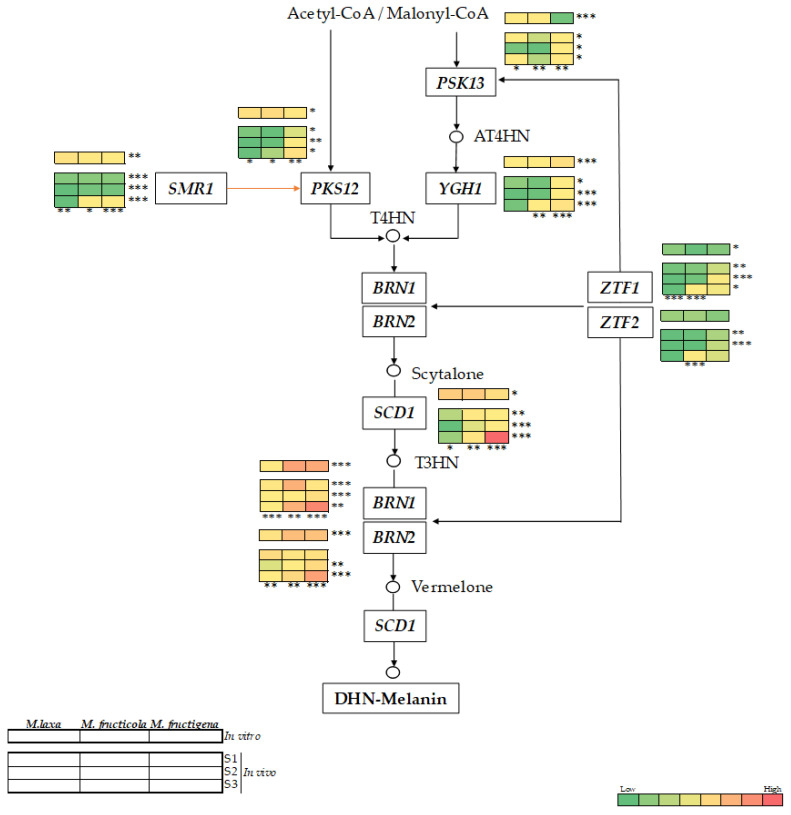
Putative DHN-melanin biosynthesis pathway of *Monilinia* spp. Black arrows indicate conserved steps between species, while the orange arrow indicates a possible difference between species. Heat maps represent the relative gene expression of each gene of the pathway for both in vitro conditions and in inoculated ‘Red Jim’ nectarines at three stages of brown rot development (S1: peel surface maceration stage; S2: colonization stage; and S3: spreading stage). The color of the heat map represents the relative gene expression intensity. For each gene, the asterisks indicate significant differences among species (right) for in vitro conditions, among species for each stage (right) and among stages for each species (bottom) in nectarines (* *p* < 0.05; ** *p* < 0.01; *** *p* <0.001). The reader is referred to Supplementary Figure S2 for a complete overview of the statistics.

The expression of the nine genes related to the DHN-melanin pathway, previously identified in the genome of the three main species of *Monilinia* spp., was analyzed both in in vitro conditions and in infected ‘Red Jim’ nectarines (Figure 5).

In the close organism *B. cinerea*, DHN-melanin biosynthesis is initiated by two polyketide synthases (*PKS12* or *PKS13*), depending on the condition in which the fungus is grown (i.e., darkness or lighting conditions). Hence, *PKS12* and *PKS13* genes are developmentally regulated and are required for melanogenesis in sclerotia/mycelium and conidia, respectively. Specifically, in *B. cinerea*, these genes are under the regulation of *SMR1* or *ZTF1/2* transcription factors, respectively [12].

Our results for *Monilinia* spp. under in vitro conditions (Figure 5) demonstrated that the transcription factor *SMR1* was clearly more activated than the transcription factors *ZTF1* and *ZTF2* for all the three species analyzed. This mechanism seemed to be conserved with *B. cinerea*, in which this pathway is activated under darkness conditions. Accordingly, the biosynthesis was putatively initiated by the *PKS12*, which was more expressed than *PKS13* in all the species, and specially in *M. fructigena*. The species *M. fructicola* expressed significantly higher levels of *PKS12* gene than *M. fructigena* (3.27-fold), and a significant increase of *PKS13* gene levels compared to *M. laxa* (1.71-fold) and *M. fructigena* (5.73-fold). In this sense, the main structure observed in *M. fructigena* cultures under in vitro conditions was mycelia; thus, the main function was probably performed by *PKS12*. In fact, in *M. fructigena*, a greater expression of *PKS12* (11.55-fold) was observed compared to *PKS13*. However, *YGH1* gene, putatively acting downstream the *PKS13* gene, was significantly more activated in *M. fructigena* than in *M. laxa* (6.14-fold) or *M. fructicola* (8.96-fold), despite the very low expression of *PKS13*. This indicates that *M. fructigena* was able to use this pathway. Interestingly, the melanin-related gene cluster analysis revealed that in the specific case of *M. fructigena*, the *PKS12* gene was not physically linked to the *SMR1* transcription factor, as did occur in *M. laxa* and *M. fructicola*, thus indicating that in this species, *PKS12* gene is not under the control of *SMR1*. Whether or not *SMR1* could somehow act by regulating the expression of *YGH1* gene specifically in this species remains to be unraveled. Only in *M. fructicola*, the *PKS13* gene is noted for being more up-regulated than in the other species, which could, in part, be related to its ability to produce conidia irrespective of the lighting condition in which it is grown. Regarding the downstream genes of the pathway, THN reductase genes (*BRN1* and *BRN2*), acting in two different points during the biosynthesis, were also highly expressed both in *M. fructicola* and in *M. fructigena* as compared to *M. laxa*, which could explain the higher melanin-like pigments produced by these two species under in vitro conditions (Figure 2B). Finally, for the *SCD1* gene, no significant differences were observed between *M. fructicola* and *M. laxa*, and only a significant higher expression occurred in *M. fructicola* compared to *M. fructigena* (2.35-fold).

Overall, the expression level of the DHN-melanin genes under in vivo conditions (Figure 5) varied depending on the species and the stages of the infection process. Both in *M. fructicola* and in *M. fructigena*, the expression level of all genes tended to increase paralleling the progression of the disease. In contrast, the expression of most genes for *M. laxa* decreased over time. These results were in line with the different melanin-like production pattern observed for the three species during its infection process (Figure 4). As for the in vitro results, the expression levels of *SMR1* transcription factor were higher compared to both *ZTF1* and *ZTF2*. The maximum melanin-like production observed at S3 coincided with the maximum expression of *SMR1*, yet this response was also observed for *ZTF1* and *ZTF2* in *M. fructicola* but not in *M. fructigena*, in which the expression levels of these transcription factors remain stable during the disease development. A similar expression pattern was observed for both *PKS12* and *PKS13* in the three *Monilinia* spp., although the expression levels were higher for *PKS12*, and especially in *M. fructigena*. The *YGH1* gene was also more activated in *M. fructigena*, and especially at the spreading stage of the disease, in which a significant increase also occurred for *M. fructicola*. Following the same pattern of the in vitro results, the downstream genes of the pathway were also, in general, more activated than the upstream genes, and a great expression was observed in *M. fructicola* and in *M. fructigena*. The lower expression levels detected in *M. laxa* could, in part, be due to the condition in which it was grown. In other organisms, such as *Colletotrichum acutatum,* it has been demonstrated that light is required to produce melanin, and also to achieve a greater virulence [17]. In our previous study [3], we demonstrated that *M. laxa* developed differently depending on the conditions in which it is grown, being highly dependent on the presence of light.

This said, both *BRN1* and *BRN2* genes increased with the disease development as did the melanin-production in *M. fructicola* and *M. fructigena* species, from S1 to S3. However, a significant decreased occurred for *M. laxa*, which was in line with the reduced melanin production observed as the pathogen infected the fruit. A similar pattern was observed for *SCD1*, fluctuating concomitantly to the melanin production in a species-dependent manner.

Finally, these results demonstrate that *Monilinia* spp. have the ability to activate the machinery related to the DHN-related melanin and that, in fact, the expression levels are positively correlated with the synthesis of this pigment along with the infection. Moreover, our findings emphasize the effect of environmental factors such as light on the different development of the species in the activation of the pathway. With these results, we have demonstrated that not all the species need melanin in the same stages of the disease, and this is translated to the different species-dependent activation patterns observed along the brown rot development.

### 3.5. Melanin Specific Genes Seem to Cooperate with Specific Genes to Induce Survival Mechanisms in Monilinia spp.

**Figure 6:**
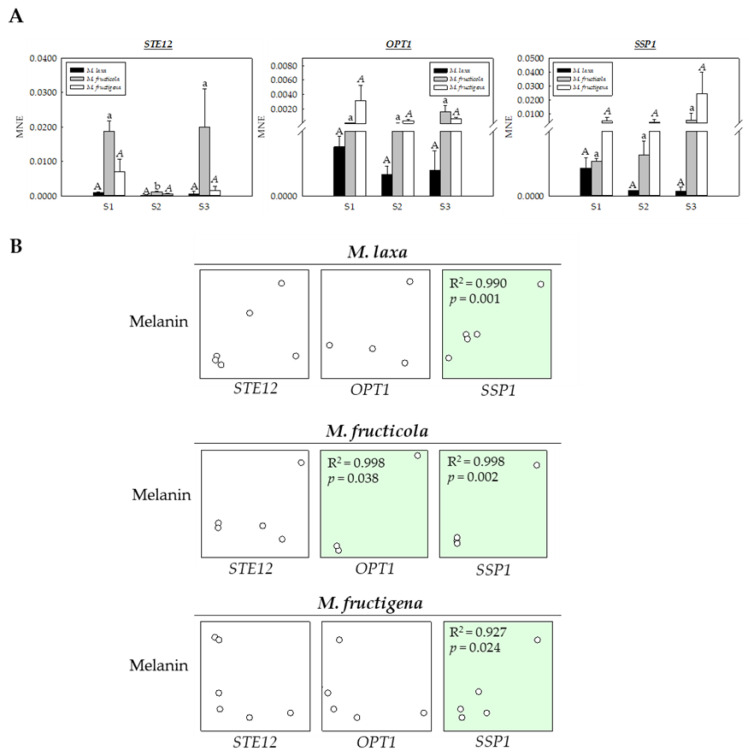
(**A**) Mean normalized expression (MNE) of *Monilinia* spp. of *STE12*, *OPT1*, and *SSP1* genes in ‘Red Jim’ nectarines infected with *M. laxa*, *M. fructicola* and *M. fructigena*, at different stages of brown rot development (S1: peel surface maceration stage; S2: colonization stage; and S3: spreading stage). Different capital, lower case, and italic letters indicate significant differences (*p* ≤ 0.05) between the stages of disease development for each *M. laxa*, *M. fructicola*, and *M. fructigena* species, respectively. Error bars represent the standard deviation of the means (n = 3). (**B**) Pearson’s product moment correlation between melanin content and *STE12*, *OPT1*, and *SSP1* gene expression in ‘Red Jim’ nectarines. Green colors denote significant (*p* ≤ 0.05) correlations.

In many fungi, melanin is not only involved in responses to stress and survival but also in morphogenic processes. To relate the capacity of the three *Monilinia* spp. to produce melanin with other related morphological and survival processes, the genes *STE12*, *OPT1*, and *SSP1*, involved in conidiation, pigmentation, melanized appressoria, and survival, and previously identified in *Monilinia* spp. [3], were selected, and their gene expression levels were analyzed in nectarines infected at three stages of brown rot development (S1, S2 and S3) (Figure 6A).

The gene expression analysis of the three selected genes revealed a different expression pattern between species but also different patterns of disease progress for each species. Specifically, in the case of *STE12* gene, involved in the formation of melanized appressoria in *M. grisea* [34], no significant differences were observed for *M. laxa* or *M. fructigena*, although a significant decrease occurred for *M. fructicola* at the S2 stage. Accordingly, the higher expression in *M. fructicola* at S1 could be explained by the appressoria formation, when the hypha adheres to the surface of the host resulting in the peel surface maceration. On the other hand, the increased expression in *M. fructicola* at S3 could be more related to the process of conidiation and pigmentation characteristic of this species, as described in a previous study with *M. grisea* [35], highlighting the importance of this gene for the infection process.

In the case of the *OPT1* gene, no significant differences were observed, neither among species nor among the three stages of brown rot development for each species. However, the expression of this gene was slightly higher in *M. fructicola* and *M. fructigena*, which could be related to the production of conidia, colony pigmentation, and/or mycelial growth under darkness [3]. Similar results were also observed in *C. gloeosporioides*, showing increased conidia production, pigmentation, and low mycelial growth under in vitro conditions, demonstrating the key role of *OPT1* [36].

Regarding the *SSP1* gene, a clear tendency, different among species, was observed, yet without significant differences. The expression levels of this gene for both *M. fructicola* and *M. fructigena* were higher compared to *M. laxa* and tended to increase as the disease progressed. In contrast, the expression levels in the case of *M. laxa* were higher at S1 and decreased as the fungus colonized the tissue.

Given these interesting differences, a correlation analysis was performed to unravel a possible relationship between melanin-like pigments production and the expression of these genes during the brown rot development (Figure 6B). Results demonstrated a positive correlative between melanin-like pigment production and *OPT1* for *M. fructicola* species (R^2^ > 0.99), but also a positive correlation between these pigments and *SSP1* gene expression for all the three *Monilinia* spp. (R^2^ > 0.92–0.99). A previous study performed by Angelini et al. [37] revealed that *SSP1* could help *M. fructicola* to survive in stressful environments. Hence, the interesting association deciphered in the present study could indicate the possible role of melanin-like pigments in mediating the survival of the *Monilinia* spp. Moreover, the different melanin production and *SSP1* expression patterns described for the different species reveal the different requirements and susceptibilities of the three *Monilinia* spp. during the infection process.

## 4. Conclusions

The results presented in this study have identified for the first time the presence of DHN biosynthetic and regulatory genes in the genome of the three *Monilinia* species. Interestingly, not all species shared the same gene location in the genome; differences were revealed with respect to *M. fructigena*, and specifically for the *SMR1* gene, indicating to a different regulatory mechanism in this species. In addition, the species that produced a greater content of melanin-like pigments under in vitro conditions were *M. fructigena* and *M. fructicola*. Under in vivo conditions, a similar pattern was observed between *M. fructigena* and *M. fructicola*, showing increased content of melanin-like pigment as the disease developed. However, *M. laxa* produced more melanin in the early stage of the disease (recognition and peel surface maceration) and this decreased throughout its development. These results highlight for the first time the different melanin requirements of *M. laxa*, *M. fructicola*, and *M. fructigena* during their infection process. In addition, a close relationship has been shown between the production of these pigments and the activation of genes related to survival, such as the *SSP1* gene. These results contribute to the better understanding of this devasting disease and, more particularly, provide evidence for the different survival and adaptative capabilities of the different species, which will allow for better designed targeted management of the disease.

## Figures and Tables

**Table 1:** Identification of DHN biosynthetic and regulatory genes from *Monilinia laxa*, *M. fructicola*, and *M. fructigena* obtained by BLAST analysis using previously described genes from *Botrytis cinerea* as query sequences. The results for *Monilinia* spp. indicate the ID of the corresponding gene, and the identity and coverage result from the BLAST analysis.

	*M. laxa*			*M. fructicola*			*M. fructigena*		
Gene	Gene ID	Identity (%)	Coverage (%)	Gene ID	Identity (%)	Coverage (%)	Gene ID	Identity (%)	Coverage (%)
***PKS12***	Monilinia__049320	87.49	99.67	MFRU_042g00180.1	87.82	99.67	g3016.t1	87.82	99.44
***PKS13***	Monilinia__009450	87.98	99.91	MFRU_053g00290.1	87.93	99.95	g3467.t1	87.42	99.91
***SCD1***	Monilinia__009400	90.42	100.00	MFRU_053g00340.1	91.62	100.00	g3472.t1	90.42	100.00
***YGH1***	Monilinia__012010	86.49	93.90	MFRU_030g00600.1	87.78	99.76	g3325.t1	83.46	96.10
***SMR1***	Monilinia__049330	85.75	96.16	MFRU_042g00190.1	86.65	99.57	g712.t1	86.80	95.10
***BRN1***	Monilinia__025870	27.15	89.62	MFRU_016g00660.1	96.20	100.00	g4002.t1	93.77	100.00
***BRN2***	Monilinia__009410	96.62	100.00	MFRU_053g00330.1	96.99	100.00	g3471.t1	93.99	100.00
***ZTF1***	Monilinia__009420	73.59	94.77	MFRU_053g00320.1	72.76	96.92	g3470.t1	71.38	96.92
***ZTF2***	Monilinia__009430	70.92	96.56	MFRU_053g00310.1	71.64	96.23	g3469.t1	70.45	89.34

## Data Availability

Not applicable.

## Supplementary Materials

The following supporting information can be downloaded at: https://www.mdpi.com/article/10.3390/jof9020138/s1, Figure S1: Mean normalized expression (MNE) of 9 genes of the DHN-melanin biosynthetic pathway of *M. laxa*, *M. fructicola* and *M. fructigena* grown for 7 days on PDA-T medium under darkness. Letters indicate significant differences (*p* ≤ 0.05) among species. Error bars represent the standard deviation of the means (n = 3). Figure S2: Mean normalized expression (MNE) of 9 genes of the DHN melanin biosynthetic pathway of *M. laxa*, *M. fructicola* and *M. fructigena* when infecting ‘Red Jim’ nectarines at different stages of brown rot development (S1, S2, and S3). Different capital, lower case and italic letters indicate significant differences (*p* ≤ 0.05) between the stages of disease development for *M. laxa*, *M. fructicola*, and *M. fructigena*, respectively. Error bars represent the standard deviation of the means (n = 3). For each gene, asterisk indicate significant differences among species for each stage (S1, S2, and S3) (* *p* < 0.05; ** *p* < 0.01; *** *p* < 0.001). Table S1: DHN-melanin biosynthetic genes from *B. cinerea* used as query sequences for the BLAST analysis in *Monilinia* spp. gene names and their corresponding gene ID are specified. Table S2: Real-time PCR primers set to assess the expression pattern of the selected genes.
