# Supplementary material for: Identification and Biosynthesis of DHN-melanin Related Pigments in the Pathogenic Fungi Monilinia laxa, M. fructicola, and M. fructigena
Source: J Fungi (Basel). 2023 Jan 19;9(2):138. doi: 10.3390/jof9020138 (PMC9962828; doi:10.3390/jof9020138)
Supplement: Supplementary file 1 [file jof-09-00138-s001.zip › jof-2127031-supplementary.pdf]

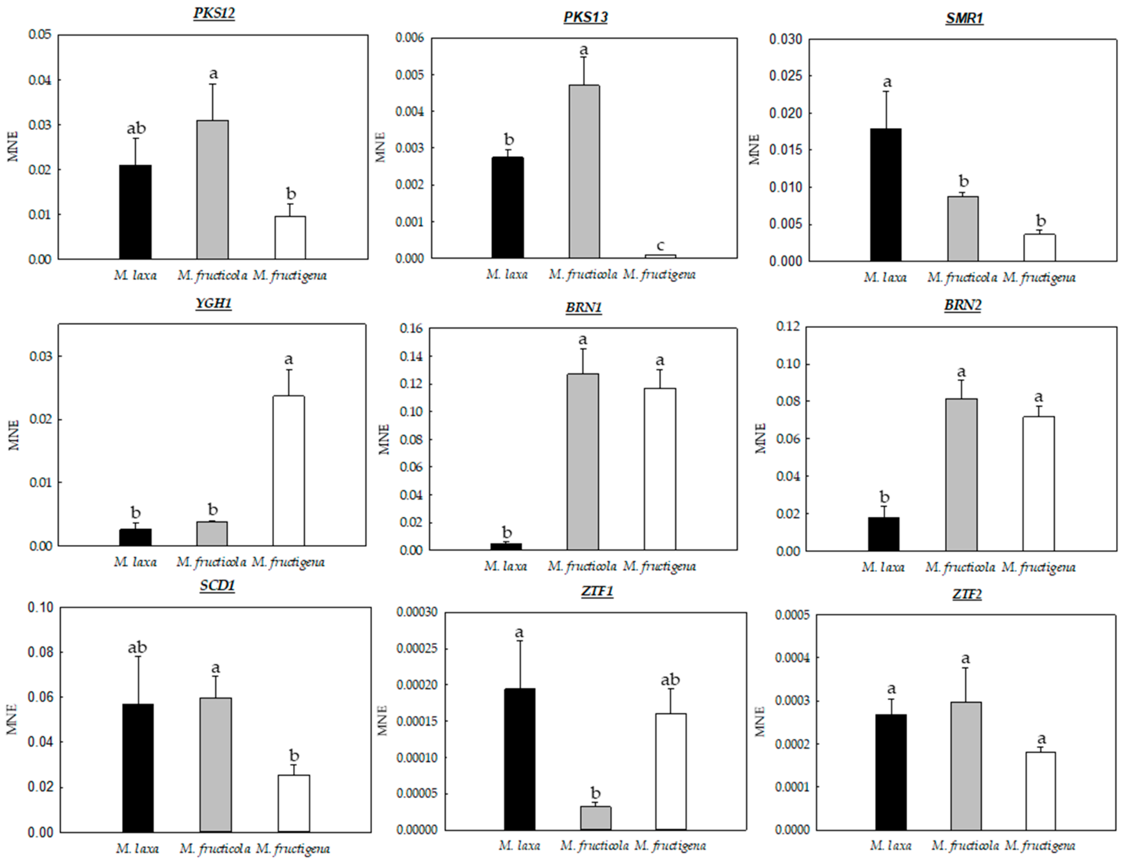

Supplementary Figure S1. Mean normalized expression (MNE) of 9 genes of the DHN-melanin biosynthetic pathway of *M. laxa, M. fructicola* and *M. fructigena* grown for 7 days on PDA-T medium under darkness. Letters indicate significant differences ($p \leq 0.05$) among species. Error bars represent the standard deviation of the means (n= 3).

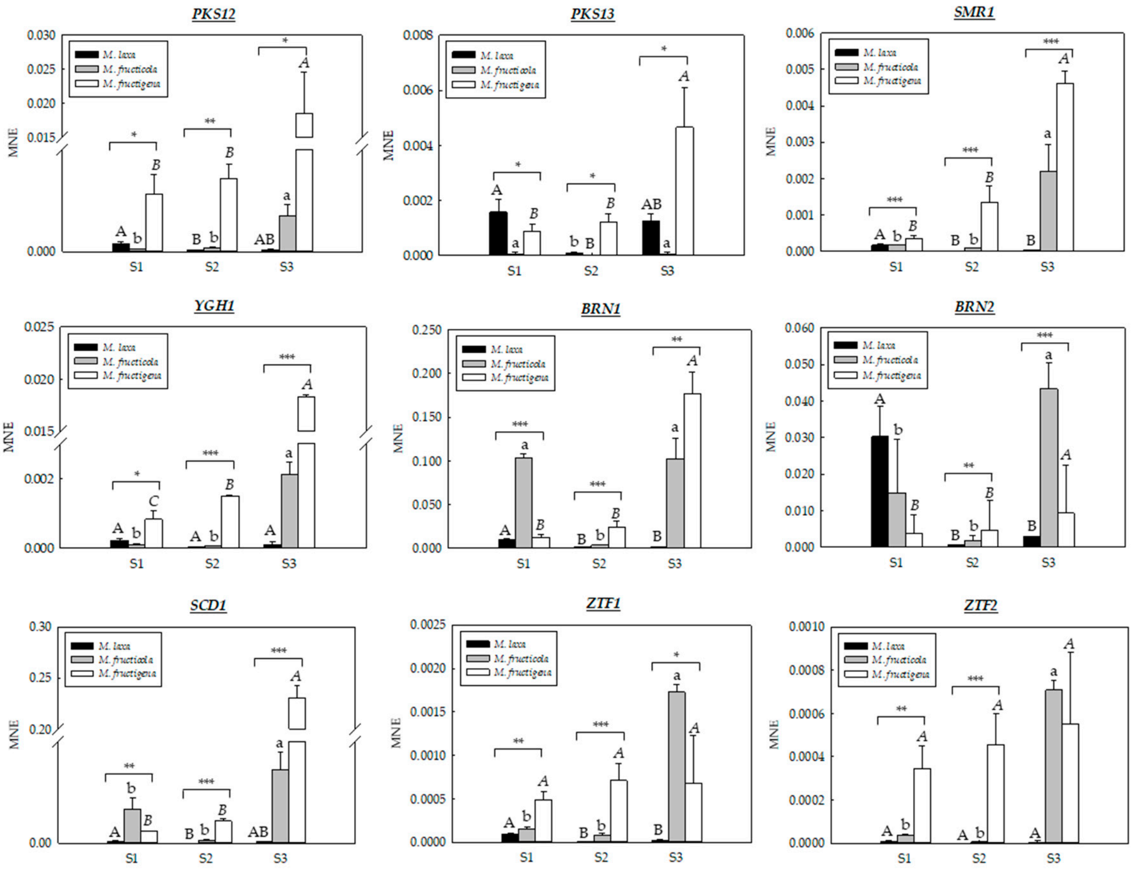

Supplementary Figure S2. Mean normalized expression (MNE) of 9 genes of the DHN melanin biosynthetic pathway of *M. laxa*, *M. fructicola* and *M. fructigena* when infecting 'Red Jim' nectarines at different stages of brown rot development (S1, S2, and S3). Different capital, lower and italic letters indicate significant differences ($p \leq 0.05$) between the stages of disease development for *M. laxa*, *M. fructicola*, and *M. fructigena*, respectively. Error bars represent the standard deviation of the means (n= 3). For each gene, asterisks indicate significant differences among species for each stage (S1, S2, and S3) (*$p < 0.05$; **$p < 0.01$; ***$p < 0.001$).

Supplementary Table S1. DHN-melanin biosynthetic genes from *B. cinerea* used as query sequences for the BLAST analysis in *Monilinia* spp. gene names and their corresponding gene ID are specified.

Gene	Full gene name	ID Query sequence
PKS12	*POLYKETIDE SYNTHASE- 12*	Bcin02g08770
PKS13	*POLYKETIDE SYNTHASE- 13*	Bcin03g08050
SCD1	*SCYTALONE DEHYDRATASE- 1*	Bcin03g08110.1
YGH1	*YELLOWISH-GREEN HYDROLASE- 1*	Bcin02g04360.1
BRN1	*BROWN 1*	Bcin03g08100
BRN2	*BROWN 2*	Bcin04g04800
SMR1	*SCLEROTIAL MELANOGENESIS REGULATORY GENE- 1*	Bcin02g08760.1
ZTF1	*TRANSCRIPTION FACTOR 1 (CLUSTERED WITH PKS12 OR PKS13)*	Bcin03g08090
ZTF2	*TRANSCRIPTION FACTOR 2 (CLUSTERED WITH PKS12 OR PKS13)*	Bcin03g08080

Supplementary Table S2. Real-time PCR primers set to assess the expression pattern of the selected genes.

Target gene	Primer name	Primer sequences (5´- 3´)	Product length (bp)	Efficiency for each species (%)	Source
PKS12	PKS12- Fw<br>PKS12- Rv	TGCTACCAACCACTCAGCAG<br>TTGGGATCAACACCCGCATT	105	*M. laxa:* 98.13<br>*M. fructicola:* 93.63<br>*M. fructigena:* 100.97	This study
PKS13	PKS13- Fw<br>PKS13- Rv	GCCTACGAGGCCATGGAAAT<br>TCGATGTCTTGAGCAGCGTT	131	*M. laxa:* 97.43<br>*M. fructicola:* 95.77<br>*M. fructigena:* 101.95	This study
SMR1	SMR1- Fw<br>SMR1- Rv	CCATTACCCGGTGGTGTTCC<br>GCATATCTGCTGGCACATGG	143	*M. laxa:* 98.55<br>*M. fructicola:* 101.57<br>*M. fructigena:* 103.36	This study
YGH1	YGH1- Fw<br>YGH1- Rv	CGAGCAGCTTGTGTCTATCG<br>TTTTGGGCTTCCCATGCCTT	98	*M. laxa:* 104.28<br>*M. fructicola:* 99.01<br>*M. fructigena:* 92.27	This study
BRN1	BRN1- Fw<br>BRN1- Rv	GGATCTGGCCAAGGTATCGG<br>ATGTCCCCTGGGACTGCTAT	158	*M. laxa:* 89.68	This study
	BRN1- Fw<br>BRN1- Rv	CCGAACCGACATGTACCACA<br>GCGACCAAGTGCAAGCATAC	98	*M. fructicola:* 90.35<br>*M. fructigena:* 90.24	This study
BRN2	BRN2- Fw<br>BRN2- Rv	CCAATATGTCCGGCGTACCA<br>AGCGATACCGTTGCAAGTGA	122	*M. laxa:* 85.14<br>*M. fructicola:* 87.51<br>*M. fructigena:* 87.81	This study
SCD1	SCD1- Fw<br>SCD1- Rv	GACTACACCACCATCGGCAA<br>CGCCGAGAAGATGTTGGGTT	121	*M. laxa:* 102.50<br>*M. fructicola:* 98.71<br>*M. fructigena:* 102.55	This study
ZTF1	ZTF1- Fw<br>ZTF1- Rv	TCCCAACAATGCTCGTCGAAT<br>GGTACGCAGCAGCCATACTC	156	*M. laxa:* 99.80<br>*M. fructicola:* 99.90<br>*M. fructigena:* 98.61	This study
ZTF2	ZTF2- Fw<br>ZTF2- Rv	TGTCGAGCTTTGGATGGCTAC<br>TGAGCCATGCGGTACATCTTA	92	*M. laxa:* 99.06<br>*M. fructicola:* 100.01<br>*M. fructigena:* 103.03	This study
STE12	STE12- Fw<br>STE12- Rv	CATCGGGCAAACACATCAGC<br>GCACTGTAATCTTCGTAGTGCG	102	*M. laxa:* 94.68<br>*M. fructicola:* 94.08<br>*M. fructigena:* 93.35	Verde-Yáñez et al. 2022
OPT1	OPT1- Fw<br>OPT1- Rv	TTCGAGCATGGGTGATAGGAC<br>CTACTCCAAGCGGGTATGCG	132	*M. laxa:* 96.34<br>*M. fructicola:* 100.01<br>*M. fructigena:* 99.29	Verde-Yáñez et al. 2022
SSP1			166		

	SSP1- Fw<br>SSP1- Rv	GGAATCCAAATGGATCGCAGTG<br>AGGACAGTCTCGGTATGCCA		*M. laxa:* 106.81<br>*M. fructicola:* 104.62<br>*M. fructigena:* 103.06	Verde-Yáñez et al. 2022
EF1-α	EF1-α-Fw<br>EF1-α-Rv	CTGAGTACCCACCTCTCGGA<br>ACCGGCCTTTTCTTGCTTCT	98	*M. laxa:* 99.3<br>*M. fructicola:* 102.13<br>*M. fructigena:* 104.41	Verde-Yáñez et al. 2022
TEF2	TEF2-Fw<br>TEF2-Rv	GGTGTGACGATGAAGAGTGATG<br>TGAAGGAGAGGGAAGGTGAAAG	129	100.40	Tong et al. 2009
